# Rationalizing mineral gypsum use through microbially enriched municipal solid waste compost for amelioration and regaining productivity potential of degraded alkali soils

**DOI:** 10.1038/s41598-023-37823-5

**Published:** 2023-07-21

**Authors:** Yash Pal Singh, Sanjay Arora, Vinay Kumar Mishra, Atul Kumar Singh

**Affiliations:** ICAR-Central Soil Salinity Research Institute, Regional Research Station, Lucknow, 226002 India

**Keywords:** Microbiology, Plant sciences, Environmental sciences, Environmental impact

## Abstract

Reclamation of alkali soils to harness their productivity potential is more complex due to the presence of excess sodium ions, poor hydraulic conductivity and infiltration rate, resulting in poor plant growth and crop productivity. Sodic soil reclamation using inorganic ameliorants like mineral gypsum or phosphogypsum is beyond the reach of small and marginal farmers having alkali soils because of their higher market prices and shortage of availability. Conjoint use of inorganic and organic amendments can be a pragmatic solution for improving soil physico-chemical and biological properties and sustaining crop productivity. Municipal solid waste compost (MSWC) available in abundant quantity if enriched with the efficient halophilic microbial consortium and used in conjunction with a reduced dose of gypsum can be a cost-effective approach for sustainable reclamation of alkali soils and harnessing their productivity potential. Hence, a field experiment was conducted on a high alkali soil (pH_2_ 9.2 ± 0.10), electrical conductivity (EC) 1.14 ±  0.12﻿ dS m^−1^, exchangeable sodium percentage 48 ± 2.50 and organic carbon (0.30%) was conducted during 2018–19 to 2020–21 to study the combined effect inorganic and organic (enriched municipal solid waste compost (EMSWC)) amendments on amelioration of alkali soils and sustaining productivity of rice–wheat cropping system. Application of gypsum @ 25% GR + enriched MSW compost @ 10 t ha^−1^ (T_6_) showed significant improvement in soil physico-chemical and biological properties over the sole application of organic (T_3_ and T_4_), inorganic (T_2_) and control (T_1_). A significant improvement in soil fertility status in terms of available nitrogen and micronutrients as well as CO_3,_ HCO_3_, Cl, Ca and Mg content were recorded with the combined application of organic and inorganic soil amendments (T_5_ and T_6_) over the sole application of mineral gypsum. Soil microbial biomass carbon (MBC), nitrogen (MBN) and phosphorus (MBP) improved significantly due to the application of EMSWC with gypsum over the application of gypsum only. Grain yield of rice and wheat increased significantly (P < 0.05) owing to the application of a reduced dose of gypsum (25% GR) and EMSWC @ 10 t ha^−1^ (T_6_) with values of 5.55 and 3.83 t ha^−1^, respectively over rest of the treatments. Three years economic analysis of the study revealed that treatments T_6_ and T_5_ gave the highest positive net return whereas it was lowest in treatment T_1_ and negative in treatment T_2_. The highest benefit-to-cost ratio (B:C) was obtained in treatments T_6_ and T_5_ which were significantly higher compared to the rest of the treatments.

## Introduction

In arid and semi-arid regions, salt accumulation in soils is a major factor in land degradation and reducing crop productivity^1^. Nearly 1000 million hectares of soil is having some degree of salinization or sodification problem around the world^2^. Most of these soils are having poor plant growth and nutrient availability due to ion toxicity and ionic imbalance, low osmotic potential, moisture in the rhizosphere, extremely poor water conductivity and low available moisture range for plants^3^. The presence of salt load in such soils also diminishes microbial activities as well as microbial biomass^4,5^. In India about 6.73 million ha is salt-affected land including salinity [dominant in chloride and sulfates of Na, Ca, and Mg, pH < 8.5; electrical conductivity (EC) > 4 dS m^−1^; sodium adsorption ratio (SAR) > 15], of which about 3.77 million ha are reported as alkali soil [dominant in carbonate, bicarbonate, and silicates of Na, pH > 8.5; exchangeable sodium percentage (ESP) > 15 and EC < 4 dS m^−1^]^6^. The amelioration of alkali lands and harnessing their productivity potential is of great importance. Gypsum (CaSO_4_·2H_2_O) is generally used as a chemical ameliorant for the reclamation of these soils. Various studies have reported that there is no improvement in soil physical and biological properties with the use of chemical amendments only because of low hydraulic conductivity^7^ and also its reduced availability in the present scenario due to its non-agriculture uses and high reclamation cost, restricts chemical reclamation. Some studies have been conducted to investigate the efficacy of organic amendments, like farm yard manure (FYM) and press mud (a by-product of sugar mills) alone and in conjunction with chemical amendments for the amelioration of alkali soils and found a significant improvement in their physical, chemical and biological properties and crop productivity^8,9^.

Disposal of municipal solid waste (MSW) generated as a by-product of industries, mining, municipality and agricultural processes is a major challenge in urban areas. About 1300 million tons of MSW are generated every year globally. By 2025, it is expected to reach to 2200 million tons annually^10^. Its disposal for land-filling contributes to polluting groundwater, breeding of insects and spreading of diseases. In India, about 62 million tons of MSW are generated per day^11^. The disposal of ever-increasing amounts of MSW is becoming a serious problem all over the world. Several strategies have been made for its effective utilization but its efficacy in the reclamation of alkali soils has not been scientifically explored. Composting of degradable MSWand its use as a source of organic matter is the simplest and best option for its management. Municipal solid waste compost (MSWC), having high organic matter content and low concentration of organic and inorganic pollutants helps in improving the physical, chemical, biochemical and microbial properties of salt-affected soils^12^*.* Application of MSWC could hasten salt leaching, reduces exchangeable sodium percentage (ESP) and electrical conductivity (EC), and increases infiltration, water holding capacity (WHC) and aggregate stability^13^. Furthermore, MSWC acts as a source of nutrients that can raise soil fertility and aid in maximising the productivity of salt-affected soils^14,15^. MSWC may act as a non-conventional carbon and nutrient source to reduce the adverse effects of alkalinity on soil properties. MSWC produced by waste treatment plants through the mechanical process are generally poor in plant nutrients therefore its adequacy in agricultural use has been reported as poor^16^. The halophilic plant growth-promoting microbes (HPGPM) have prospective for bio-amelioration of salt-affected soils^17^ by enhancing productivity and these require a suitable carrier for field application. Thus, enrichment of mechanically processed MSWC with HPGPM will help in improving the quality of compost. Given the nutritional importance and quality of the compost, the present study was therefore conducted to enrich the MSWC with halophilic plant growth-promoting microbes and its utilization in conjunction with inorganic amendments to ameliorate alkali soils and regaining the productivity potential of alkali soils under rice–wheat cropping system.

## Methods and materials

### Site characterization

#### Location

Field experiments were conducted at ICAR-Central Soil Salinity Research Institute, Regional Research Station, Research Farm, Lucknow, India located at 26° 47′ 58″ N latitude and 80° 46′ 24″ E longitude and 120 m above mean sea level (AMSL). Taxonomically the experimental soil of the farm was classified as Typic Natrustalfs, sandy loam to clay loam in texture^18^. The soil was poor in physical properties and nutrient status because of poor soil water and soil air relations caused by higher bulk density (> 1.6 g cm^−3^) and poor hydraulic conductivity. The farm land is gently sloping in NE to 97.6 m in the SW direction within contours of 99.0 m.

#### Climate

**Figure 1:**
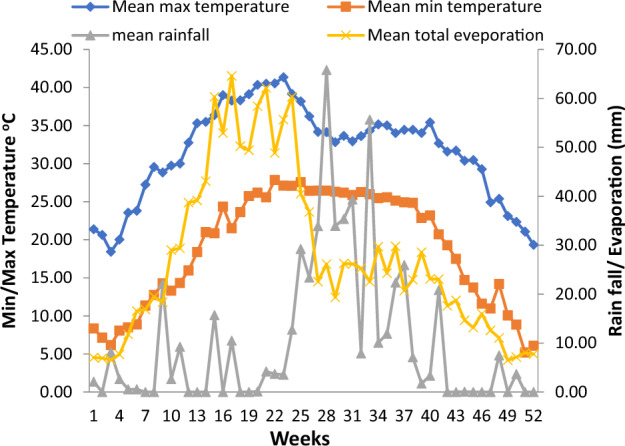
Climatic features of the experimental site. The given figures are the average of 10 years of weather data (2010–2020).

The climate of the experimental site is semi-arid, subtropical and monsoonal with an annual average rainfall of 817 mm. The maximum rainfall received in between 23 and 40 standard weeks (June–October) was 394 mm, which is 91% of the total annual rainfall. The remaining 9% was received between 41 and 22 standard weeks (November–May). The annual average evaporation rate (1580 mm) varies with increasing air temperature and atmospheric water demands increased gradually from 1 to 22 weeks (January–June). During the rainy season (June–October) evaporation rate gradually decreases following monsoonal rains. Further, it decreased gradually in the month of December due to reduced temperature. During the rainy season (June–September), the farm land remains in water surplus and in the remaining period (November–May) there is water deficit due to less precipitation and a higher evaporation rate. The mean maximum temperature (39 °C) was recorded in June and mean minimum temperatures (7.1 °C) in January. The mean annual temperature during the study period was recorded as 24.6 °C (Fig. 1).

#### Initial soil properties

The soil samples were collected from the experimental site at a soil depth of 0–15 cm with an auger. A part of the sample was air dried, grounded in a pestle and mortar passed through a 2.0 mm sieve and the other part was kept in the refrigerator for microbial and bio-chemical analysis. The processed samples were analysed for physico-chemical and biological properties. The different fractions of soil viz. sand, silt and clay contents were determined following International Pipette Method^19^. For bulk density determination, soil was extracted with an intact core sampler of 10 cm in diameter and 15 cm in length^20^, the infiltration rate in soil was estimated using double concentric infiltrometer cylinders of 60 cm outer and 30 cm inner diameters, and the soil porosity was calculated as (1 − Bulk Density/Particle Density) × 100^21^. For the determination of the cation exchange capacity (CEC) of the soil sample, the ammonium acetate-sodium acetate substitution method was employed^21^. The soil pH and electrical conductivity (EC) were determined in 1:2 soil:water solution using a glass-electrode digital pH meter (Systronics Type 361) and TDS-conductivity meter (Systronics Type 306), respectively. Exchangeable sodium concentration (cmol kg^−1^)/cation exchange capacity (cmol kg^−1^) × 100 was used for computing the exchangeable sodium percentage (ESP) of the soil. Soil organic carbon (SOC) content was analyzed by Walkley and Black’s rapid titration wet-oxidation method^22^. Available N, P and K content in processed soil samples was estimated by distillation with KMnO_4_ and NaOH^23^, sodium bicarbonate extraction method^24^ and sodium acetate extraction method^25^, respectively. The ionic concentration of Na and K in soil saturation extract were measured using Flame Photometry (Systronics, Type 128), while Ca and Mg ions by the Versenate titration method^26^. However, the carbonate (CO_3_^−^) and bi-carbonate (HCO_3_^−^) ion concentration were determined by titration with 0.01 N H_2_SO_4_ using phenolphthalein and methyl orange indicators. To optimize the gypsum dose for the experimental field soil, the mineral gypsum (CaSO_4_·2H_2_O) requirement (GR) was determined using the modified method as described by Schoonover^27^. To analyse the biological properties of the soil, the usual serial dilution plate count method was used on nutrient agar, potato dextrose agar, and actinomycetes-specific media, for enumerating the respective bacterial, fungal, and actinomycetes populations^28^, respectively. To analyse the soil microbial biomass carbon (MBC) and nitrogen (MBN) content, CHCl_3_ fumigation extraction technique^29^ was used. The soil microbial biomass phosphorus (MBP) was measured by the chloroform fumigation extraction method^30^. Soil biochemical properties including urease and dehydrogenase activities were determined using phosphate buffer and urea substrate^31^ and triphenyl tetrazolium chloride (TTC) method^32^. The initial properties of the experimental soil are presented in Table 1.

**Table 1:** Initial properties of soil of the experimental field.

Soil properties	0–15 cm	Soil properties	0–15 cm
Sand, %	65.55	Ca^++^ (mel^−1^)	2.2
Silt, %	18.5	Mg^++^, mel^−1^	2.4
Clay, %	16.0	Na^+^, mel^−1^	60.34
Textural class	Loam	K^+^, mel^−1^	2.29
Bulk density, g cm^−3^	1.59	CO_3_, mel^−1^	3.1
Soil porosity, %	42.4	HCO_3_, mel^−1^	1.8
Infiltration rate, mm day^−1^	2.1	Cl, mel^−1^	4.8
CEC, cmol (p +) kg^−1^ soil	12.40	Bacterial count, cfu g^−1^	1.3 × 10^6^
Gypsum requirement (G.R.), Mg ha^−1^	10.0	Fungal count, cfu g^−1^	0.2 × 10^5^
pH (1:2)	9.20	MBC, mg kg^−1^	115.5
EC_2_, dS m^−1^	1.14	MB-N, mg kg^−1^	1.20
Exchangeable Na percentage (ESP)	48	MB-P, mg kg^−1^	0.03
Soil organic C, %	0.30	Urease, µg urea g ^−1^ h^−1^	100.4
Available N, kg ha^−1^	142.5	Dehydrogenase activity, µg TPF g^−1^ day^−1^	84.3
Available P, kg ha^−1^	22.12		
Available K, kg ha^−1^	286.5		

*EC* electrical conductivity, *MBC* microbial biomass carbon, *MBN* microbial biomass nitrogen, *MBP* microbial biomass phosphorus.

### Imposition of treatments

**Figure 2:**
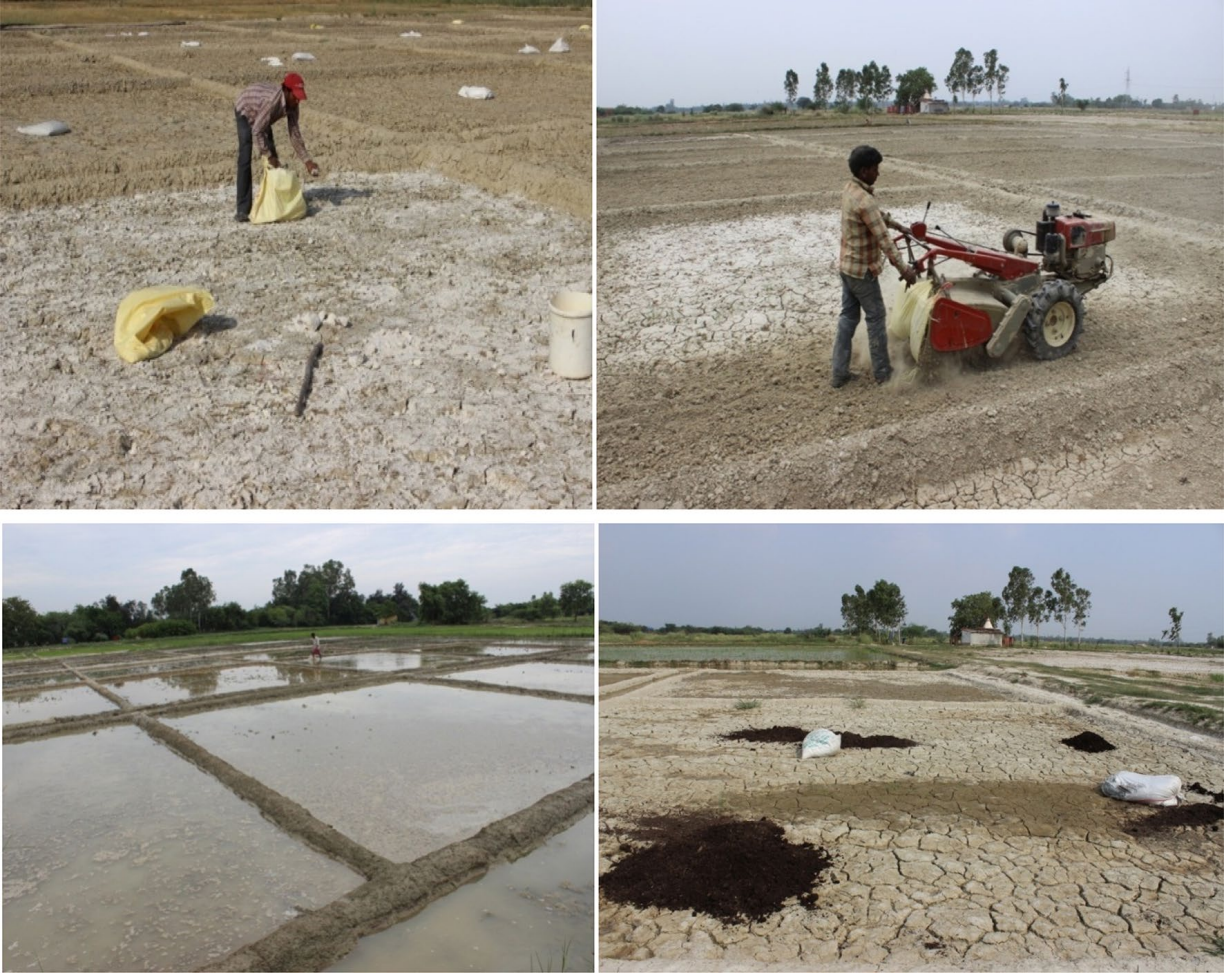
Layout plan and imposition of treatments in the experimental field.

**Figure 3:**
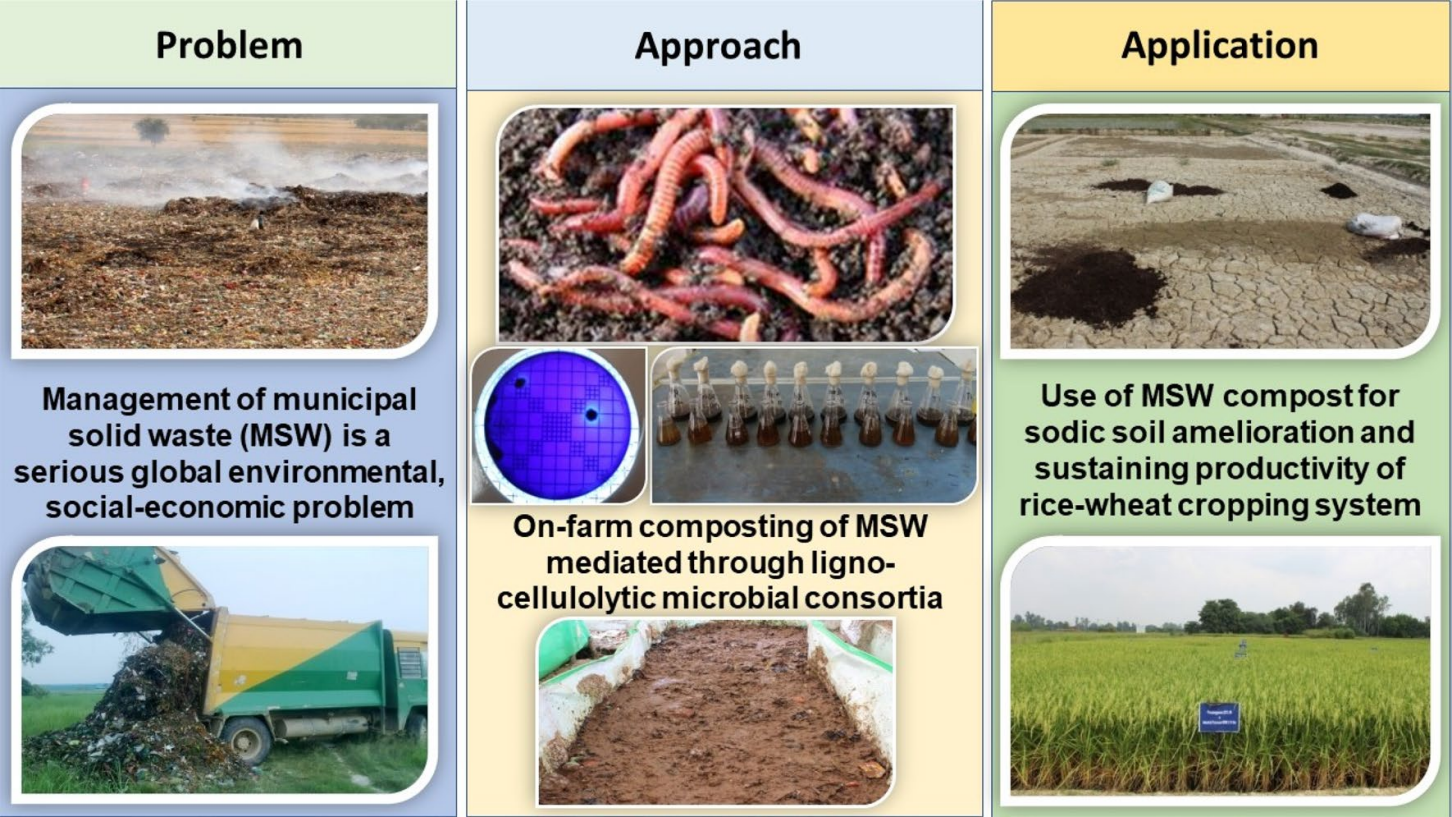
The approach adapted for on-farm composting of MSW and its utilization for crop production in sodic soils to rationalize gypsum amendment.

To quantify the dose of mineral gypsum to be applied in each treatment, its chemical composition was analysed using standard procedures. The mineral gypsum (CaSO_4_·2H_2_O) constituted 18.3% Ca and 16.1% sulphur. The calculated amount of gypsum was broadcasted as per treatment during the month of June in the upper surface soil layer (up to 10–12 cm depth) and mixed with a power tiller (Fig. 2). About 10 cm of water was ponded for at least 10 days to displace the NaSO_4_ as the reaction product of Ca–Na exchange beyond the root zone as per the standard sodic soil reclamation protocol. As per treatment, organic sources of amendments like un-enriched MSW compost and enriched MSW compost were applied @ 10 t ha^−1^ and mixed in 15 cm of surface soil. The approach adapted for on-farm composting of MSW and its utilization for crop production in sodic soils to rationalize gypsum amendment is presented in Fig. 3.

### Experimental design and treatment details

A field experiment on sodic soil was conducted constituting six treatments viz. T_1_—control (no soil amendment), T_2_—gypsum @ 50% GR, T_3_—un-enriched MSW compost @ 10 t ha^−1^, T_4_—enriched MSW compost @ 10 t ha^−1^, T_5_—gypsum @ 25% GR + un-enriched MSW compost @ 10 t ha^−1^, T_6_—gypsum @ 25% GR + enriched MSW compost @ 10 t ha^−1^ during 2018–19 to 2020–21 with rice-wheat crops in a Randomized block design and the treatments replicated four times. The reclamation protocol including field bunding, levelling, gypsum application, and leaching of salts was strictly followed before the application of organic amendments in the treatments.

Salt-tolerant varieties of rice ‘CSR36’ and wheat ‘KRL210’ were grown for three years as a test crop in the rainy and winter seasons, respectively. The recommended dose of fertilizers (150 kg N:60 kg P_2_O_5_:40 kg K_2_O:25 kg ZnSO_4_ ha^−1^) was applied uniformly in both the crops through urea, diammonium phosphate (DAP), muriate of potash (MOP) and zinc sulphate. Half the quantity of nitrogenous fertilizer and a full dose of phosphorus, potash, and zinc were applied as basal uniformly in all the treatments. The remaining 50% of N fertilizer was applied in two equal splits at 30 days after transplanting (DAT) of rice and sowing of wheat and at panicle initiation stages in both crops. Rice seedlings of salt tolerant variety ‘CSR 36’ was transplanted at 20 × 15 cm row-to-row and plant-to-plant spacing in the second week of July and harvested in the first week of November. A salt-tolerant variety of wheat ‘KRL 210’ was sown after harvesting of rice in the month of November in the same treatments and harvested in April. Other crop management practices as and when required were remain the same in all the treatments. Grain and straw yields of both crops were calculated after harvesting from each net plot area (8 m × 7 m) and expressed in t ha^−1^.

The tube well used for irrigation of the experimental field was situated about 100 m away from the experimental field. Before using this water for irrigation its quality was analysed in the laboratory following standard methods. It has low electrolyte concentration and the corresponding EC values ranged between 0.78 and 0.90 dS m^−1^. Among the cations, Na dominates over Ca and Mg followed by K and the anions like, bicarbonates plus carbonates dominates over calcium, while sulphates were absent. This water was having residual alkalinity to the tune of 1.3–1.5 meq l^−1^ which was harmless for irrigating the crops grown in salt-affected soils.

### Enrichment of MSW compost

The MSW compost for this study collected from nearby municipal solid waste treatment plant was enriched with consortia of efficient halophilic plant growth-promoting microbes that includes *Azotobacter*, *Phosphobacteria* and zinc solubilizing *Bacillus* sp. bacteria. The microbially enriched MSW compost was prepared as Compost enricher blocks for easy transportation and adoption by farmers for field application. It was analyzed for bio-chemical and microbiological properties after stabilization. The bio-chemical composition of the enriched and unenriched MSW compost is given in Table 2.

**Table 2:** Bio-chemical composition of enriched and unenriched MSW compost.

Parameters	Values	
	Un-enriched MSW compost ± SD	Enriched MSW compost ± SD
Bulk density (g cm^−3^)	0.78 ± 0.01	0.89 ± 0.01
Moisture (%)	9.23 ± 0.21	11.12 ± 0.16
pH (1:5)	7.60 ± 0.24	7.86 ± 0.32
EC (dS m^−1^)	0.64 ± 0.26	1.22 ± 0.24
CEC [cmol (p+) kg^−1^ compost]	186 ± 8.0	183 ± 11.0
Total C (%)	25.47 ± 0.32	20.38 ± 0.26
Total N (%)	0.64 ± 0.06	0.79 ± 0.04
C:N ratio	39.80 ± 0.40	25.79 ± 0.35
Total P (%)	0.41 ± 0.04	0.71 ± 0.04
Total K (%)	0.57 ± 0.02	0.84 ± 0.04
Total Ca (mg kg^−1^)	340.00 ± 26.2	406.00 ± 21.3
Total Mg (mg kg^−1^)	195.00 ± 26.5	228.00 ± 23.5
Total Zn (mg kg^−1^)	1620.00 ± 45.6	2115.00 ± 51.2
Bacterial population (cfu g^−1^)	48 × 10^5^ ± 4.0	75 × 10^5^ ± 6.0
Fungal population (cfu g^−1^)	45 × 10^5^ ± 5.0	79 × 10^5^ ± 4.0
Phosphate solubilizing microbes (cfu g^−1^)	9.0 × 10^5^ ± 3.0	53 × 10^5^ ± 5.0
Total Ni (mg kg^−1^)	49.12 ± 2.3	42.6 ± 4.1
Total Pb (mg kg^−1^)	29.30 ± 4.0	24.3 ± 5.0
Total Cd (mg kg^−1^)	7.30 ± 0.23	5.12 ± 0.11
Total Cr (mg kg^−1^)	45.50 ± 1.2	40.23 ± 1.3

*EC* electrical conductivity.

### Post-harvest soil analysis

Soil samples collected from 0 to 15 cm soil depth from all the treatment plots, after three years of the experiment. Samples were air dried in shade, grounded through wooden pestle and sieved through 2 mm sieve for the analysis of chemical properties following the methods used to analyse initial soil properties. Part of the soil samples (about 100 g) was stored in refrigerator at 4 °C in polyethene bags for the analysis of soil biological properties.

### Statistical analysis

The generated data was analysed statistically employing MSTAT-C version 2.1. To test the significance (p < 0.05) of treatment effect, the least significant difference (LSD) test at 5% significance level^33^ probability was used. The analysed data are presented as an average value of four replications with standard error (±).

## Results

### Bio-chemical properties of enriched and un-enriched MSW compost

Bio-chemical configuration of the enriched and un-enriched MSW compost is given in Table 2. Most of the bio-physical and chemical properties of the compost improved after enrichment with microbial formulations. The bulk density and moisture content after enrichment increased to the level of 14.10 and 20.48%, respectively. The data revealed that there was slight increase in pH and EC of the compost, but not much variation was observed in cation exchange capacity (CEC) between enriched and un-enriched composts. A significant reduction in C/N ratio (54.32%) was observed after enrichment of MSW compost. Nutrient status including total P, K, Ca, Mg and Zn increased substantially with the enrichment of MSW compost. The population of bacterial, fungal and phosphate solubilizing bacteria increased remarkably with the enrichment of MSW compost. The heavy metal contents decreased with microbial enriched compost.

### Effect of enriched compost on soil properties

#### Physical properties

The data given in Table 3 showed that the soil bulk density at surface (0–15 cm) and sub-surface (15–30 cm) layer reduced significantly when organic amendments were applied in combination with inorganic amendments over the control (T_1_) and application of only inorganic amendment (T_2_). Maximum drop in bulk density value was noticed in treatment T_6_ where EMSWC and reduced dose of gypsum (25% GR) was applied combinedly. Minimum bulk density was observed in control (T_1_) and T_2_ treatment (Table 3). Treatment T_6_ (Gypsum @ 25% GR + EMSWC @ 10 t ha^−1^) also exerted significant improvement in hydraulic conductivity with a value of 0.077 cm h^−1^ over treatment T_1_ (0.046 cm h^−1^) and T_2_ (0.055 cm h^−1^). This was closely followed by T_4_ (Enriched MSW compost @ 10 t ha^−1^) and T_5_ (Gypsum @ 25% GR + un-enriched MSW compost @ 10 t ha^−1^). The infiltration rate increased by 224, 89 and 356% with the application of EMSWC (T_6_) over T_1_, T_2_ and initial values, respectively (Table 3).

**Table 3:** Effect of organic and inorganic amendments on physical properties of soil.

Treatments	Bulk density (g cm^−3^)		Hydraulic conductivity (cm h^−1^)	Infiltration rate (mm day^−1^)
	0–5 cm	5–10 cm		
T_1_: control (no amendments)	1.57 ± 0.01	1.58 ± 0.01	0.046 ± 0.001	6.52 ± 0.23
T_2_: gypsum @ 50% G.R	1.53 ± 0.02	1.56 ± 0.01	0.055 ± 0.002	11.15 ± 1.13
T_3_: un-enriched MSW compost @ 10 t ha^−1^	1.52 ± 0.01	1.54 ± 0.02	0.055 ± 0.001	15.12 ± 2.12
T_4_: enriched MSW compost @ 10 t ha^−1^	1.51 ± 0.01	1.55 ± 0.01	0.075 ± 0.001	17.50 ± 0.11
T_5_: gypsum @ 25% G.R. + un-enriched MSW compost @ 10 t ha^−1^	1.48 ± 0.02	1.53 ± 0.02	0.075 ± 0.001	18.66 ± 0.22
T_6_: gypsum @ 25% G.R. + enriched MSW compost @ 10 t ha^−1^	1.46 ± 0.02	1.51 ± 0.01	0.077 ± 0002	21.12 ± 1.12
Initial	1.59 ± 0.01	1.62 ± 0.02	0.008 ± 0.001	4.63 ± 0.12
LSD (P = 0.05)	0.03	0.13	0.011	1.12

*LSD* least significant difference.

#### Chemical properties

Soil analysis data presented in Table 4 demonstrated that the use of EMSWC along with a lower dose of gypsum decreased soil pH, electrical conductivity (EC), and exchangeable sodium percentage (ESP) while increasing soil organic carbon (SOC). The combined use of reduced gypsum and EMSWC (T_6_) recorded significant reduction in soil pH with a value of 8.5 over the treatment T_1_, T_3_, and T_4_ but at par with T_2_, and T_5_. Similarly, EC and ESP with values of 0.47 dS m^−1^ and 35 reduced significantly with treatment T_6_over the treatment T_1_, T_3_, and T_4_ and at par with T_2_, and T_5_. Reduced quantity of gypsum along with either un-enriched or enriched MSW compost improved SOC content in alkali soils as compared to only use of gypsum. Maximum improvement in SOC with a value of 3.90 g kg^−1^ was recorded in treatment T_6_ which was significantly higher over rest of the treatments and showed 25.80, 21.87, 21.87, 14.70 and 5.40% over T_1_, T_2_, T_3_, T_4_, and T_5_, respectively. Available N content under different treatments varies from 123.5 to 155.2 kg ha^−1^. The effect of EMSWC on available N content is shown in Table 4. The highest improvement in available N (155.2 kg ha^−1^) was recorded in treatment T_6_ which was significantly higher over rest of the treatments. The treatments in which EMSWC and gypsum were used in combined mode reported higher available P content than the application of gypsum alone. Plots treated with reduced dose of gypsum and EMSWC (25%G.R. + EMSWC) resulted 43 and 36% increase in available P over control (T_1_) and 50% G.R. (T_2_), respectively. Similarly, available K increased by 33 and 14% in treatment T_6_ over T_1_ and T_2_, respectively. Data given in Table 4 revealed more increment in Mg^2+^ and K^+^ contents in the soil extract in treatment T_6_ than rest of the treatments whereas, maximum reduction in Na^+^ and increment in Ca^2+^ were recorded in treatment T_2_.

**Table 4:** Effect of organic and inorganic amendments on soil chemical properties after three years of amendment and cultivation of rice–wheat cropping system.

Treatments	T_1_	T_2_	T_3_	T_4_	T_5_	T_6_	LSD (P = 0.05)
pH (1:2)	9.1 ± 0.02	8.6 ± 0.01	9.0 ± 0.01	9.0 ± 0.02	8.7 ± 0.01	8.5 ± 0.01	0.21
EC_2_ (dS m^−1^)	0.83 ± 0.11	0.49 ± 0.12	0.70 ± 0.11	0.56 ± 0.13	0.48 ± 0.12	0.47 ± 0.11	0.10
ESP	47 ± 1.52	38 ± 1.23	42 ± 2.12	42 ± 2.11	40 ± 1.23	35 ± 1.42	3.36
OC (g kg^−1^)	3.10 ± 0.12	3.20 ± 0.11	3.20 ± 0.11	3.40 ± 0.11	3.70 ± 0.12	3.90 ± 0.11	0.14
Available N (kg ha^−1^)	123.5 ± 2.23	144.6 ± 2.36	140.2 ± 3.12	141.3 ± 2.26	153.3 ± 3.11	155.2 ± 3.12	2.23
Available P (kg ha^−1^)	16.60 ± 2.10	17.45 ± 1.53	18.23 ± 2.22	18.06 ± 1.63	21.20 ± 2.21	23.75 ± 2.11	1.51
Available K (kg ha^−1^)	246.1 ± 4.63	287.2 ± 4.52	301.3 ± 3.26	315.6 ± 6.23	327.6 ± 2.22	332.7 ± 2.22	8.23
Ca^++^ (meq l^−1^)	3.62 ± 0.01	6.20 ± 0.12	3.75 ± 0.11	4.20 ± 0.11	4.62 ± 0.12	5.75 ± 0.11	0.54
Mg^++^ (meq l^−1^)	3.00 ± 0.11	3.75 ± 0.06	3.37 ± 0.23	3.75 ± 0.12	5.35 ± 0.11	5.37 ± 0.26	1.12
Na^+^ (ppm)	296.16 ± 2.63	248.86 ± 4.23	289.81 ± 3.86	296.16 ± 4.52	271.66 ± 6.12	269.28 ± 4.23	12.21
K^+^ (ppm)	3.14 ± 0.001	2.74 ± 0.012	2.44 ± 0.013	3.21 ± 0.012	3.21 ± 0.012	4.37 ± 0.011	0.46

The values are mean ± SE of four replications.

T_1_—control (no amendments), T_2_—gypsum @ 50% G.R., T_3_—un-enriched MSW compost @ 10 t ha^−1^, T_4_—enriched MSW compost @ 10 t ha^−1^, T_5_—gypsum @ 25% G.R. + un-enriched MSW compost @ 10 t ha^−1^, T_6_—gypsum @ 25% G.R. + enriched MSW compost @ 10 t ha^−1^.

*pH*_*2*_* and EC*_*2*_ soil and water suspension ratio of 1:2, *OC* organic carbon, *ESP* exchangeable sodium percentage, *LSD* least significant difference.

#### Biological properties

The data furnished in Table 5 showed that the microbial population swelled with the application of organic amendments. The population of bacteria and fungi increased remarkably in soils where mineral gypsum and EMSW compost were used in a conjoint mode and the salt tolerant varieties of rice–wheat were grown. The highest bacterial population (8.46 × 10^4^ cfu g^−1^) was enumerated in treatment T_6_ (gypsum @ 25% GR + EMSWC @ 10 t ha^−1^) and it was followed by the treatment T_5_, T_4_ and T_3_ and the lowest in T_1_. Similar trend was observed in case of fungal population. Application of enriched MSW compost as organic amendment along with mineral gypsum increased fungal population about 98% over the only application of gypsum (Table 5). Similarly, actinomycetes’s population increased significantly with the conjoined use of enriched MSW compost and rational dose of gypsum. However, microbial populations were unstable during the cropping season. The highest content of microbial biomass carbon (MBC), microbial biomass N (MBN) and microbial biomass phosphorus (MBP) were observed in soil from treatment T_6_ followed by T_5_, and T_4_. In our study urease and dehydrogenase activities in the soil enhanced significantly over the control, sole use of inorganic amendments and initial values in all of the treatments that combined the application of organic and inorganic amendments. Maximum increase in these parameters was observed in treatment T_6_ (Table 5).

**Table 5:** Effect of enriched municipal solid waste compost on soil microbial properties.

Treatments	Bacteria (cfu g^−1^ soil)	Fungi (cfu g^−1^ soil)	Actinomycetes (cfu g^−1^ soil)	MBC (mg kg^−1^)	MBN (mg kg^−1^)	MBP (mg kg^−1^)	Urease (µg urea g ^−1^ h^−1^)	Dehydrogenase (µg TPF g^−1^ day^−1^)
T_1_: control (no amendments)	2.88 × 10^4^	44.5 × 10^2^	61.2 × 10^3^	115.6	1.51	0.11	155.2	105.3
T_2_: gypsum @ 50% G.R	4.05 × 10^4^	45.5 × 10^2^	69.6 × 10^3^	145.2	2.32	0.26	170.5	112.6
T_3_: un-enriched MSW compost @ 10 t ha^−1^	5.04 × 10^4^	56.5 × 10^2^	71.1 × 10^3^	221.3	5.06	0.32	210.3	121.6
T_4_: enriched MSW compost @10t ha^−1^	5.67 × 10^4^	60.5 × 10^2^	145.8 × 10^3^	226.2	6.12	0.41	226.5	132.0
T_5_: gypsum @25%G.R. + un-enriched MSW compost @10 t ha^−1^	8.27 × 10^4^	60.0 × 10^2^	234.9 × 10^3^	256.3	7.52	0.56	240.9	137.4
T_6_: gypsum @ 25% G.R. + enriched MSW compost @10t ha^−1^	8.46 × 10^4^	90.5 × 10^2^	374.4 × 10^3^	288.5	9.07	1.23	263..8	157.6

### Micronutrients and heavy metals content in soil

After three years of field experiments with rice–wheat cropping system, heavy metal contents like Co, Cr and Pb and micronutrients like Cu, Fe, Mn and Zn in the soil were analyzed. The analysed data given in Table 6, showed that the concentration of Co and Cr declined in treatments where both organic and inorganic amendments were applied whereas, a marginal increase in Pb content was observed over the initial value but it was considerably below the permissible limit^34,35^. The micronutrient contents like Cu, Fe, and Zn increased over the initial value in soil representing treatments where combined use of organic and inorganic amends was made. Highest increase in iron (Fe) content was resulted in treatment T_4_ where industrial processed MSW compost was applied in conjunction with mineral gypsum.

**Table 6:** Combined effect of inorganic amendments and enriched municipal solid waste composton heavy metal and micronutrient contents in soil surface (0-15 cm).

Treatments	Co (ppm)	Cr (ppm)	Pb (ppm)	Cu (ppm)	Fe (ppm)	Mn (ppm)	Zn (ppm)
T_1_: control (no amendments)	0.667	0.065	0.135	3.72	21.12	7.12	0.57
T_2_: gypsum @ 50% G.R	0.651	0.065	0.132	3.22	20.36	6.70	0.63
T_3_: un-enriched MSW compost @10 t ha^−1^	0.507	0.063	0.146	2.98	25.96	6.92	1.13
T_4_: enriched MSW compost @10t ha^−1^	0.467	0.059	0.134	2.90	28.53	6.60	1.05
T_5_: gypsum @ 25%G.R. + un-enriched MSW compost @ 10 t ha^−1^	0.463	0.069	0.167	2.56	22.96	4.25	1.05
T_6_: gypsum @ 25% G.R. + enriched MSW compost @ 10 t ha^−1^	0.432	0.064	0.178	2.32	20.36	3.89	1.13
Initial	0.760	0.070	0.113	3.18	13.64	7.62	0.77
LSD (P = 0.05)	0.04	ns	0.003	0.34	1.12	1.11	0.04

*LSD* least significant difference, *ns* non-significant.

### Effect of organic and inorganic amendments on crop growth and yield

Plant height of rice and wheat crop was non-significantly affected due to either sole application of inorganic or combined use of organic and inorganic amendments but it was greatest (124.0 cm and 83.55 cm) in treatment T_2_ and T_6_, respectively. Number of productive tillers per rice hill, which is a major yield contributing character and is precisely related to panicle density and grain yield influenced by the application of EMSWC with gypsum. Number of productive tillers hill^−1^ recorded with treatment T_6_ were significantly higher over treatment T_1_, T_3_ and T_4_ but statistically at par with T_5_ and T_6_. Similar pattern was observed in panicle density, panicle length and dry matter content of rice and wheat. Whereas, highest spikelet fertility was recorded in treatment T_2_ which was significantly higher over T_1_, T_3_ and T_4_ but at par with T_5_ and T_6_. Numbers of grains per panicle were also significantly higher in treatment T_6_ but it was at par with T_5_. Although 1000 grain weight was maximum in treatment T_2_ but there was no significant difference in this parameter between the treatments. Highest rice grain yield was observed in treatment T_6_ that was at par with T_2_ and T_5_ but significantly better than T_1_, T_3_ and T_4_ (Table 7).

**Table 7:** Combined effect of inorganic amendments and enriched municipal solid waste compost on crop growth of rice and wheat.

Treatments	Rice				Wheat		
	Plant height (cm)	Productive tillers hill^−1^	Panicle density (m^2^)	Dry matter (g hill^−1^)	Plant height (cm)	Spike density (m^2^)	Dry matter (g hill^−1^)
T_1_: control (no amendments)	111.22	9.65	286.0	93.59	69.2	363.41	647.3
T_2_: gypsum @ 50% G.R	124.40	14.77	387.9	144.55	77.9	388.22	674.6
T_3_: un-enriched MSW compost @ 10 t ha^−1^	123.43	11.65	345.0	123.63	76.12	341.30	623.3
T_4_: enriched MSW compost @ 10 t ha^−1^	122.35	12.80	359.1	130.43	76.22	343.90	632.3
T_5_: gypsum @ 25% G.R. + un-enriched MSW compost @ 10 t ha^−1^	123.40	13.42	381.0	144.55	77.90	391.31	681.4
T_6_: gypsum @ 25% G.R. + enriched MSW compost @ 10 t ha^−1^	123.40	16.47	389.6	148.10	83.55	394.20	688.3
LSD (P = 0.05)	ns	3.12	11.23	6.35	ns	6.23	8.63

*LSD* least significant difference, *ns* non-significant.

Yield attributing characters like spike length, grains per spike, and 1000-grain weight of wheat were significantly higher in treatment T_6_ compared to the treatment T_1_, T_3_, and T_4_ but statistically at par with the treatments T_2_ and T_5_. Grain yield of wheat in treatment T_6_ was 110.75% and 20.61% higher over treatment T_1_ and T_2_, respectively (Table 8). Enrichment of MSW compost with microbes enhanced 21.36% wheat grain yield over un-enriched MSW compost used along with the mineral gypsum (Table 8).

**Table 8:** Effect of organic and inorganic amendments on yield attributes and yields of rice and wheat crops.

Treatments	Rice					Wheat			
	Panicle length (cm)	Spikelet fertility (%)	Grains panicle^−1^	1000 grain weight (g)	Grain yield (t ha^−1^)	Length of spike (cm)	Grains spike^−1^	1000 grain weight (g)	Grain yield (t ha^−1^)
T_1_	21.30	73.2	114.30	22.22	4.21	13.08	30.95	32.0	1.86
T_2_	25.37	87.5	132.22	26.62	5.27	18.78	33.70	41.8	3.25
T_3_	23.52	76.3	126.02	24.62	4.50	16.85	30.95	38.5	2.43
T_4_	23.77	81.2	129.62	23.55	4.70	17.20	32.20	30.4	2.47
T_5_	25.37	85.3	138.05	25.37	5.17	18.05	33.50	40.9	3.23
T_6_	25.52	85.8	138.30	25.95	5.45	19.55	35.10	43.1	3.92
LSD P = 0.05)	0.53	5.23	5.32	ns	0.43	0.63	3.12	2.13	0.21

T_1_—control (no amendments), T_2_—gypsum @ 50% G.R., T_3_—un-enriched MSW compost @ 10 t ha^−1^, T_4_—enriched MSWcompost @ 10 t ha^−1^, T_5_—gypsum @ 25% G.R. + un-enriched MSW compost @ 10 t ha^−1^, T_6_—gypsum @ 25% G.R. + enriched MSW compost @ 10 t ha^−1^.

*LSD* least significant difference.

### Cost economics of reclamation technology

**Figure 4:**
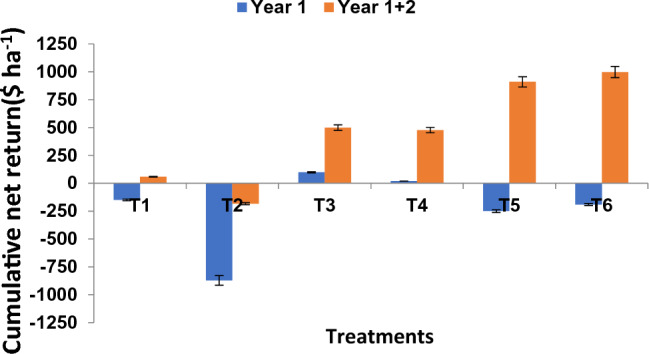
Cumulative net returns of rice–wheat cropping system over two-years and a combined use of inorganic amendments and EMSWC. T_1_—control (no amendments), T_2_—gypsum @ 50% G.R., T_3_—un-enriched MSW compost @ 10 t ha^−1^, T_4_—enriched MSW compost @ 10 t ha^−1^, T_5_—gypsum @ 25% G.R. + un-enriched MSW compost @ 10 t ha^−1^, T_6_—gypsum @ 25% G.R. + enriched MSW compost @ 10 t ha^−1^.

During first year of reclamation, a small positive net return was computed in treatments T_3_ and T_4_ where only organic amendments were used, whereas, it was negative in the rest of the treatments. In treatment T_2_, highest negative net return was obtained that was followed by treatment T_5_ and T_6_. This was obviously due to higher cost involved in soil reclamation with gypsum applied @ 50% GR and 25% GR, respectively. However, during second year, when no amendment was applied and the crop productivity under treatment T_6_ and T_5_ was comparatively higher, the highest positive net return was obtained under treatment T_6_ followed by T_5_ and the lowest in treatment T_1_, while, negative return was still there in treatment T_2_. This demonstrates that even after the second year of cultivation, the expense of reclamation with the addition of gypsum is not recouped (Fig. 4). According to cost-economic analysis, treatment T_6_ had the greatest B/C ratio, which was comparable to treatment T_5_ but much higher than the other treatments.

## Discussion

### Chemical properties of enriched compost

After analysis of enriched and un-enriched MSW compost, it was observed the total nitrogen content in enriched MSW compost in comparison to un-enriched MSW compost increased, while total carbon content decreased. This may be due to considerable reduction in weight during decomposition of organic matter particularly of total C through C loss in respiration as CO_2_ after enrichment with microbial formulations as compared to loss in total N content in the un-enriched compost. Similar finding related to increased total N and decrease in total C content per unit of material during decomposition of different organic wastes were given by previous researchers^36,37^. Singh and Kalamdhad^38^ has also reported a reduction in heavy metal content by microbial inoculants because of enhancing enzymatic activities during composting process. The microorganisms are able to absorb, detoxification and transformation of heavy metals that resulted to immobilize metals in the solid wastes^39,40^.

### Combined effect of inorganic amendment and enriched MSW compost on soil physico-chemical properties

The present study was undertaken with the aim to evaluate the potential of microbial enriched municipal solid waste compost for amelioration of alkali soils and monitor its effect on rice–wheat crop productivity. Sole application of gypsum, while being successful in significantly improving soil chemical properties, does not have much effect on soil physical and biological properties. Inability of gypsum to improve soil physical and biological properties has been earlier reported by several workers^41–43^. Application of Gypsum @ 25% GR + EMSWC @ 10 t ha^−1^ (T_6_) also exerted significant improvement in hydraulic conductivity with a value of 0.077 cm h^−1^ over control (T_1_) and application of gypsum treatment @ 50% G.R. (T_2_) with the values of 0.046 cm h^−1^ and 0.055 cm h^−1^, respectively. This was closely followed in treatment T_4_ and T_5_ in which only enriched MSW compost @ 10 t ha^−1^ and combined use of gypsum @ 25% GR + un-enriched MSW compost @ 10 t ha^−1^ were applied, respectively. Significant increase in infiltration rate in treatment T_6_ over T_1_, T_2_ and initial value may be because of improvement in pore geometry and transmission pores resulting from addition of organics^44,45^. Significant reduction in soil pH, EC, ESP and increased organic carbon content due to application of EMSWC composts in combination with reduced dose of gypsum (T_6_) over rest of the treatments may be attributed to increase in the organic carbon content, improved biological or enzymatic activities, producing more root biomass^45–48^ and higher evolution of CO_2_ and production of organic acids in soil which reduces the redox potential of soil thereby increasing the replacement of Na^+^ to Ca^2+^^17,49–52^. Significant improvement in SOC and ESP due to application of organic materials in salt affected soils has also been reported^53^. In our study, available N, P, and K content in soil from treatment T_6_ was significantly higher over rest of the treatments. This might be due to constant release of N from EMSW compost and creation of organic acids and mineralization products during decomposition which solubilizes the insoluble compounds that enhances the availability of P in soil^51,54^. Enhanced soil microbial and enzymatic activities vis-à-vis decomposition of organic matter resulted in mineralization of N and K in soil. Also, similar trend has been observed in case of Mg^2+^ and K^+^ contents whereas, maximum reduction in Na^+^ and increment in Ca^2+^ were recorded in treatment T_2_. This may be because of synergistic effect of gypsum and organic source of amendments^48,55^.

### Combined effect of inorganic amendment and enriched MSW compost on soil microbial and biochemical properties

Remarkable increase in soil microbial population with combined application of mineral gypsum and EMSWC over the initial population and rest of the treatments was observed in the present study. This may be due to the improvement of soil microbial activities caused from substantially higher availability of substrate from the combined use of organic and inorganic amendments compared to application of only gypsum as soil amendment^48,56,57^. Similarly, fungal population increased with application of EMSWC in combination with gypsum increased to the tune of about 98% over the application of gypsum only. This showed that applying both organic and inorganic amendments together resulted in a greater improvement in soil microbial flora than sole application of inorganic amendments. According to Walmsley and Cerdà’s^58^, organic matter is the main influence on soil characteristics and the abundance of macrofauna in citrus plantation soils. The population of actinomycetes has also increased significantly when enriched MSW compost was applied in combination with lower dose of mineral gypsum. This is due to addition of organic sources of amendments. However, microbial populations were unstable during the cropping seasons.

Microbial biomass carbon (MBC), microbial biomass N (MBN) and microbial biomass phosphorus (MBP) in soil amended with EMSWC and reduced dose of gypsum (25% GR) (treatment T_6_) were higher due to enhanced soil mineralization as a result of increased soil microflora and associated with increase in CO_2_ release thereby resulting in soil aeration and stimulates enzymatic activities^59^. The huge increase in soil microbial activity caused by the addition of organic amendments in alkali soils is attributable to the large amounts of easily accessible energy sources^60^. Organic amendments augment the amount of substrate available to the microbial population and also reduce the impact that pH and osmotic pressure have on the organisms^61^. The addition of organic amendments to alkali soil improved soil fertility directly by increasing soil enzymes and their activity^14^. In the current study, urease activity substantially increased over the control and initial urease activity status in the soil in the treatments where organic amendments were added along with gypsum. Treatment T_6_ showed the greatest improvement in urease activities as a result of the addition of organic fractions, which may contain intracellular and extracellular enzymes that promote microbial activity in the soil^62,63^. In comparison to the control and the use of only inorganic amendments, soil dehydrogenase activities also increased after the addition of EMSWC with gypsum supplements and the cultivation of rice and wheat. The availability of high quantity of organic matter added to the soil that acts as substrate for the microorganisms, may be attributed to the increased microbial and enzymatic activities^42,64^.

### Combined effect of inorganic amendment and enriched MSW compost on micronutrients and heavy metal contents in soil

Organic amendments application through EMSWC with lesser quantity of gypsum reduced the concentration of heavy metals like Co and Cr whereas, the Pb concentration increased over the initial content in soil but it was below the permissible limit^35,65^. The increase in Pb content was obtained in soil sample from the treatment where industrial processed MSW was added. This may be due to supplemented with industrial processed MSW compost procured from solid waste treatment plant. The status of micronutrient contents viz. Cu, Fe, and Zn increased with the combined usage of EMSWC and gypsum in alkali soils.

### Combined effect of inorganic amendment and enriched MSW compost on Crop growth and yield

Three years average data revealed that addition of both organic and inorganic amendments had significant effect on growth and yield of rice as well as wheat crops. This is because of significant improvement in the physical, chemical and biological properties of soil after organic matter decomposition in EMSWC and subsequently increases organic acid exudates in the soil that mobilizes dissolution of soil calcium and reduces soil pH and ESP and increases soil organic carbon content^66,67^ resulting in increased plant growth. By enhancing the physical and chemical conditions of the rhizosphere, the addition of organic matter to the soil has been shown to have positive impacts on crop root growth^68^. This might be because organic amendments, which contain humic acid and other physiologically active compounds like amino acids, have improved biological activity in crop rhizosphere^69^. Yield and yield attributes in alkali soils are significantly affected because of restricted water movement, nutrient translocation, and toxic effect of sodium salt in the rhizosphere. However, addition of organic matter through EMSWC improved physico-chemical and biological properties of soil resulting in the improved crop growth, related yield attributes and crop yields^68,70^. The use of EMSWC aided in the drainage of surplus salts to a deeper layer, decreased the salt concentration in topsoil, which benefited plant growth, and ultimately boosted crop yields^71,72^.

## Conclusions

Significant improvement in C:N ratio and microbial properties was observed after enrichment of MSW compost. Addition of enriched MSW compost @ 10 t ha^−1^ along with the reduced dose of gypsum showed significant effect on soil physico-chemical and microbial properties over the sole application of organic and inorganic amendments. The soil fertility status in terms of available N, P and K was favourably influenced with combined use of EMSWC and gypsum. Application of EMSWC significantly influenced the crop growth, yield attributes and yields. Maximum rice and wheat grain yields were recorded with conjunctive use of enriched MSW and gypsum that was significantly higher over the only use of either organic and inorganic amendments. The cost economic analysis of the study resulted the highest positive net return with treatment T_6_ whereas; negative net return was calculated with the application of inorganic amendment. This shows that reclamation cost with addition of gypsum @ 50% GR was not recovered even after second year of cultivation. The highest B/C ratio was also observed with combined use of EMSWC + gypsum @ 25% GR. Hence, it can be concluded from the study that the amelioration of alkali or sodic soil and crop yields were significantly influenced by the application of enriched MSW compost with inorganic amendment and the saved amount of mineral gypsum can be utilized for ameliorating double the salt affected area having such soil degradation problem.

## Data Availability

The datasets of the raw data are available with the corresponding author and shall be made available on reasonable request.
